# Identification of genomic regions and diagnostic markers for resistance to aflatoxin contamination in peanut (*Arachis hypogaea* L*.*)

**DOI:** 10.1186/s12863-019-0734-z

**Published:** 2019-03-12

**Authors:** Bolun Yu, Dongxin Huai, Li Huang, Yanping Kang, Xiaoping Ren, Yuning Chen, Xiaojing Zhou, Huaiyong Luo, Nian Liu, Weigang Chen, Yong Lei, Manish K. Pandey, Hari Sudini, Rajeev K. Varshney, Boshou Liao, Huifang Jiang

**Affiliations:** 1 Key Laboratory of Biology and Genetic Improvement of Oil Crops, Ministry of Agriculture, Oil Crops Research Institute of Chinese Academy of Agricultural Sciences, Wuhan, China; 2 International Crops Research Institute of the Semi-Arid Tropics (ICRISAT), Hyderabad, India

**Keywords:** Peanut, Resistance, Aflatoxin, *Aspergillus flavus*, QTL, Diagnostic marker

## Abstract

**Background:**

Aflatoxin contamination caused by *Aspergillus flavus* is a major constraint to peanut industry worldwide due to its toxicological effects to human and animals. Developing peanut varieties with resistance to seed infection and/or aflatoxin accumulation is the most effective and economic strategy for reducing aflatoxin risk in food chain. Breeding for resistance to aflatoxin in peanut is a challenging task for breeders because the genetic basis is still poorly understood. To identify the quantitative trait loci (QTLs) for resistance to aflatoxin contamination in peanut, a recombinant inbred line (RIL) population was developed from crossing Zhonghua 10 (susceptible) with ICG 12625 (resistant). The percent seed infection index (PSII), the contents of aflatoxin B_1_ (AFB_1_) and aflatoxin B_2_ (AFB_2_) of RILs were evaluated by a laboratory kernel inoculation assay.

**Results:**

Two QTLs were identified for PSII including one major QTL with 11.32–13.00% phenotypic variance explained (PVE). A total of 12 QTLs for aflatoxin accumulation were detected by unconditional analysis, and four of them (*qAFB1A07* and *qAFB1B06.1* for AFB_1_, *qAFB2A07* and *qAFB2B06* for AFB_2_) exhibited major and stable effects across multiple environments with 9.32–21.02% PVE. Furthermore, not only *qAFB1A07* and *qAFB2A07* were co-localized in the same genetic interval on LG A07, but *qAFB1B06.1* was also co-localized with *qAFB2B06* on LG B06. Conditional QTL mapping also confirmed that there was a strong interaction between resistance to AFB_1_ and AFB_2_ accumulation. Genotyping of RILs revealed that *qAFB1A07* and *qAFB1B06.1* interacted additively to improve the resistance to both AFB_1_ and AFB_2_ accumulation. Additionally, validation of the two markers was performed in diversified germplasm collection and four accessions with resistance to aflatoxin accumulation were identified.

**Conclusions:**

Single major QTL for resistance to PSII and two important co-localized intervals associated with major QTLs for resistance to AFB_1_ and AFB_2_. Combination of these intervals could improve the resistance to aflatoxin accumulation in peanut. SSR markers linked to these intervals were identified and validated. The identified QTLs and associated markers exhibit potential to be applied in improvement of resistance to aflatoxin contamination.

**Electronic supplementary material:**

The online version of this article (10.1186/s12863-019-0734-z) contains supplementary material, which is available to authorized users.

## Background

Peanut or groundnut (*Arachis hypogaea* L.) is an oilseed crop with global importance, grown in more than 100 countries with a global production of 47.53 Mt. from an area of 20.46 Mha [1]. As an excellent and cheap source of nutrition, peanuts supply abundant nutrients to the human such as proteins, lipids, carbohydrates, vitamins, minerals and fiber [2]. However, aflatoxin contamination caused by *Aspergillus flavus* and/or *Aspergillus parasiticus* is an enormous threat to peanut industry and food safety. Aflatoxins including aflatoxin B_1_, B_2_, G_1_ and G_2_ (AFB_1_, AFB_2_, AFG_1_ and AFG_2_) are highly toxic and carcinogenic substances and hard to be eliminated from contaminated materials [3–5]. Peanut tend to be infected by *A. flavus* covering the whole industrial chain including pre-harvest, during harvest, post-harvest drying, in storage and during transport [6–8]. A lot of prevention strategies for aflatoxin contamination have been implemented, including using bio-control agents, taking good agricultural practices and planting resistant varieties [9–12]. Development of peanut varieties with suitable resistance to *A. flavus* infection and/or aflatoxin production is considered to be the most effective and economical approach. However, breeding for resistance to aflatoxin is still a challenging task for breeders due to poor unavailability of highly resistance germplasm and understanding the genetics. Furthermore, the trait phenotyping faces high environmental influence and variable soil microbiome across environments and locations.

Quantitative trait locus (QTL) mapping is a conventional method to investigate the genetic basis of complex traits. In recent years, numerous QTLs have been identified in peanut for several important traits such as plant height [13], pod shape, seed shape [14, 15], drought tolerance [16] and resistances to late leaf spot [17], bacterial wilt [18] and rust [19]. Molecular markers closely linked to QTLs can be identified, validated and deployed in marker-assisted breeding. The rust resistance was successfully improved in three early maturing elite varieties using four markers linked to a major QTL [19]. However, limited efforts have been made in identifying QTLs for complex traits in peanut such as aflatoxin contamination. Six QTLs for resistance to *A. flavus* invasion were detected in three independent recombinant inbred line (RIL) populations with 6.2–22.7% phenotypic variation explained (PVE) [20], but so far, no QTL for aflatoxin accumulation has been reported in peanut. Therefore, it is necessary to identify QTLs for resistance to both *A. flavus* infection and/or aflatoxin accumulation in order to accelerate the process of peanut breeding by bringing together favorable alleles.

Aflatoxin contamination is a result of interactions among host plant, toxicogenic fungi and environment, but these factors are always inconsistent and unpredictable in field. Additionally, there is a significant G × E interaction for aflatoxin contamination, which increased the difficulties of revealing the resistance mechanism [12]. Considering minimization of environmental impact, artificial inoculation of seeds with toxicogenic *A. flavus* in laboratory is more suitable for QTL analysis comparing with field inoculation.

In our previous study, a RIL population was developed from a cross involving a susceptible peanut variety, Zhonghua10 and a resistant germplasm line, ICG 12625, and a high-density linkage map was constructed. This genetic linkage map contains 1219 loci (1175 SSR markers and 42 transposon markers) covering A and B sub-genome and all 20 chromosomes of peanut genome with map length of 2038.75 cM. The A sub-genome contains 583 loci with map length of 1010.95 cM, while the B sub-genome contains 636 loci with map length of 1027.80 cM [13]. In present study, the phenotypic data including the percent seed infection index (PSII), aflatoxin B_1_ (AFB_1_) and aflatoxin B_2_ (AFB_2_) contents of 140 individuals were collected in three consecutive years via inoculation with *A. flavus* in laboratory. QTLs for PSII, AFB_1_ and AFB_2_ contents were identified, and the genetic relationship between resistance to AFB_1_ and AFB_2_ contents was investigated by conditional QTL analysis. The obtained information would get insights on the genetic basis of resistance to aflatoxin contamination in peanut.

## Methods

### Plant materials

A mapping population consisting of 140 RIL lines was developed form a cross between Zhonghua 10 and ICG 12625 using single seed decent method. The female parent Zhonghua 10 (*A. hypogaea var. vulgaris*) is a susceptible variety to aflatoxin contamination developed by Oil Crops Research Institute of Chinese Academy of Agricultural Science (OCRI-CAAS), Wuhan, China. The male parent ICG 12625 (PI 497597, *A. hypogaea var. aequatoriana*) is a resistant germplasm line received from the International Crop Research Institute for the Semi-Arid Tropics (ICRISAT), Hyderabad, India. The RIL population (F_4_-F_6_) and the two parents were planted in experimental field of OCRI-CAAS in Wuhan, China, using a random block design with three replications in consecutive years from 2013 to 2015. Each plot contained one row, with 10–12 plants in each row, 10 cm between plants within each row and 30 cm between the rows. Field management followed the standard agricultural practices.

### Phenotyping for *A. flavus* infection and aflatoxin accumulation

The toxicogenic *A. flavus* strain (AF2202) isolated from peanut was maintained in 20% glycerol (− 80 °C) at CAAS-OCRI, China. Conidia of AF2202 were taken from the stored sample and cultured on fresh potato dextrose agar medium at 29 ± 1 °C for 7 days. Conidia were then collected and suspended in sterile water containing 0.05% Tween-80. The concentration of conidia in the suspension was determined using a haemocytometer.

About 20 g healthy and mature peanut seeds from each line were selected and surface sterilized with 75% ethanol for 1 min followed by three washes with sterile distilled water. Then, 1 ml conidial suspension (2 × 10^6^ conidia/ml) of *A. flavus* was added to peanut seeds in a sterile Petri plate. The plates were incubated at 29 ± 1 °C in dark.

The external seed infection was measured by visual inspection using the percent seed infection index (PSII), which was investigated at 7 days after inoculation. Based on previous studies [21], the invasion level of *A. flavus* was defined and classified with minor modifications as Level 0 when no conidium observed on the seed surface; Level 1 when less than 1/3 of the seed surface covered by conidia; Level 2 when 1/3–2/3 of the seed surface covered by conidia; Level 3 when more than 2/3 of the seed surface covered by conidia. The formula $\frac{\left(\mathrm{n}1+\mathrm{n}2\times 2+\mathrm{n}3\times 3\right)}{\mathrm{n}\times 3}\times 100\%$ was used to calculate the PSII, where n, n1, n2 and n3 are the number of seeds in total, level 1, level 2 and level 3, respectively.

After investigation of PSII, the peanut seeds were rinsed with 75% ethanol to remove conidia of *A. flavus* on the seed surface, and then dried at 110 °C for 60 min. Aflatoxin in these seeds were extracted by 55% ethanol solution and analyzed by high-performance liquid chromatography to detect the contents of AFB_1_ and AFB_2_ as described by Wang et al. [22].

### Statistical analysis and QTL mapping

Statistical analyses for the phenotypic data of PSII, contents of AFB_1_ and AFB_2_ were performed with SPSS Statistics 22.0 statistical software [23]. The broad-sense heritability for each trait was calculated as: H^2^ = σ2 g/(σ2 g + σ2 ge/n + σ2 e/rn), where σ2 g is genetic variance, σ2 ge is the interaction variance between genotype and environment, σ2 e is the residual (error) variance, r is the number of replications in each environment and n is the number of environments. The variance of each component was estimated by restricted maximum likelihood (REML) method as previous study described [13]. Correlation coefficients were estimated between each pair of the three traits. Genotype data was collected and the linkage map was constructed in previous study [13].

QTL mapping was conducted by composite interval mapping method in the Windows QTL Cartographer 2.5 software using mean value of each trait in each environment [24]. The default model (model 6) was selected in the software. The number of control markers, window size and walk speed were set as 5, 10 and 2 cM, respectively. The threshold of LOD for declaring the presence of a QTL was determined by 1000 permutation tests.

Conditional analysis was also performed by Windows QTL Cartographer 2.5 software based on conditional phenotypic values y(AFB_1_|AFB_2_) and y(AFB_2_|AFB_1_), which were calculated by the mixed-model method using QGA Station 1.0 software [25].

## Results

### Phenotypic evaluation of resistance to aflatoxin contamination

**Fig. 1:**
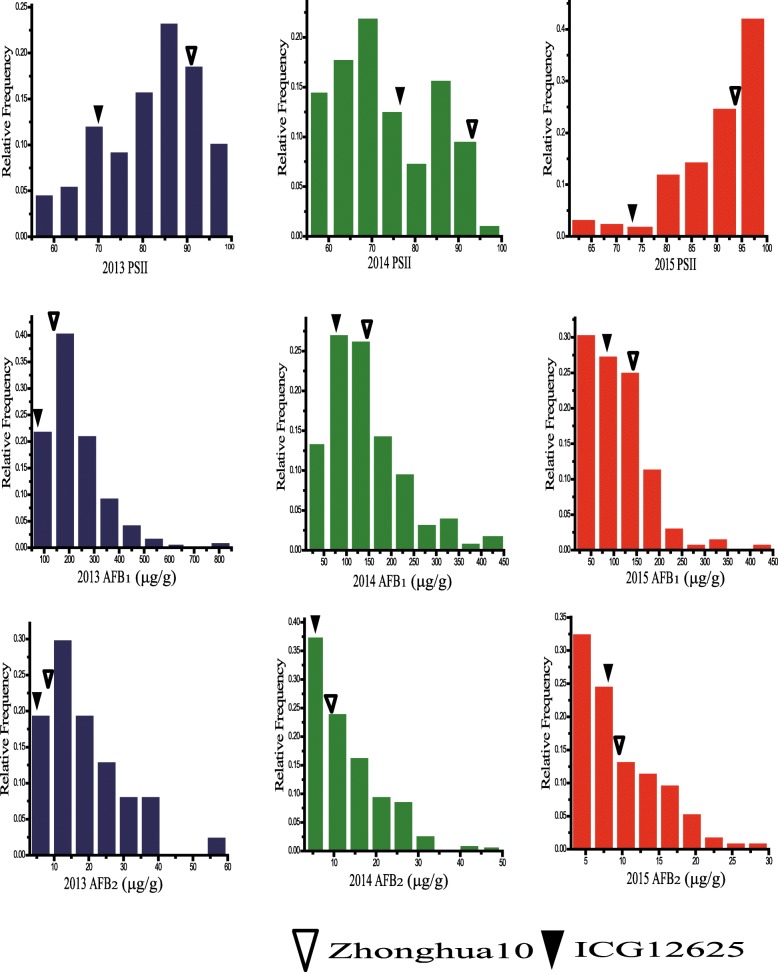
Phenotypic observation and distribution of PSII, AFB_1_ and AFB_2_ in parents and RIL population. Phenotypic distribution of PSII, AFB_1_ and AFB_2_ in RIL population across three environments. The *y-axis* represented frequency, while *x-axis* represented values of each trait. *PSII* percent seed infection index, *AFB*_*1*_ aflatoxin B_1_ content, *AFB*_*2*_ aflatoxin B_2_ content

The resistance performance of two parents and the RIL population was investigated by artificial inoculation with toxicogenic *A. flavus* in laboratory across three environments. Significant differences of PSII and aflatoxin content between Zhonghua 10 and ICG 12625 were observed (Table 1 and Fig. 1). ICG 12625 exhibited desirable resistance with lower infection rate and less aflatoxin accumulation (Table 1). Transgressive segregation and continuous distribution in the RIL population for both PSII and aflatoxin contents were observed in all the environments (Table 1 and Fig. 1), suggesting that both the parents had favorable alleles for resistance to aflatoxin contamination. Broad-sense heritability was estimated to be 0.64 for PSII, 0.78 for AFB_1_ content and 0.75 for AFB_2_ content (Table 1), indicating these traits were controlled by genetic factors. Variance analysis across the three trials also revealed that the genetic, environmental effects and genotype by environment interaction significantly affected PSII and aflatoxin contents (see Additional file 1).

**Table 1:** Phenotypic variations of PSII, AFB_1_ and AFB_2_ of two parents and RILs in three trials

Trait	Env	Parents		RIL Population			
		Zhonghua10	ICG 12625	Range	Mean ± SD	CV	*H* ^*2*^
PSII (%)	2013	92.33 ± 1.89	70.00 ± 3.74^**^	25.00–100.00	95.28 ± 15.01	0.16	0.64
	2014	93.00 ± 2.16	77.67 ± 0.47^**^	40.00–97.50	67.08 ± 14.14	0.21	
	2015	93.00 ± 1.41	73.33 ± 2.49^**^	53.33–100.00	90.35 ± 8.99	0.10	
AFB_1_ (μg/g)	2013	144.10 ± 35.10	85.39 ± 9.77	29.04–812.94	211.09 ± 124.76	0.59	0.78
	2014	143.06 ± 17.07	77.56 ± 4.43^**^	10.34–443.47	144.59 ± 83.37	0.58	
	2015	133.58 ± 14.14	86.52 ± 16.99^*^	15.68–409.87	108.96 ± 66.94	0.61	
AFB_2_ (μg/g)	2013	7.60 ± 0.48	6.40 ± 1.48	2.54–56.17	18.64 ± 11.15	0.60	0.75
	2014	8.33 ± 0.35	6.59 ± 0.59^*^	1.59–46.49	12.13 ± 8.59	0.71	
	2015	7.63 ± 0.60	6.12 ± 0.93	0.68–27.18	8.54 ± 5.70	0.67	

*PSII* percent seed infection index, *AFB*_*1*_ aflatoxin B_1_ content, *AFB*_*2*_ aflatoxin B_2_ content, *Env* environment, *SD* standard deviation, *CV* coefficient of variation, *H*^*2*^ broad-sense heritability; ^*^Difference is significant at *p* < 0.05 level, ^**^Difference is significant at *p* < 0.01 level

Pairwise correlation analyses on PSII, AFB_1_ and AFB_2_ contents across three environments showed significant positive correlation (R^2^, 0.81–0.91) (*P* ≤ 0.001) between AFB_1_ and AFB_2_ contents across environments (Table 2). But neither AFB_1_ nor AFB_2_ content was correlated with PSII (Table 2), suggesting that resistance to *Aspergillus* infection and aflatoxin accumulation were independently regulated in peanut.

**Table 2:** Correlation analysis of PSII and aflatoxins contents in RIL population

Environment	Trait	PSII	AFB_1_	AFB_2_
2013	PSII	1		
	AFB_1_	0.17	1	
	AFB_2_	0.08	0.81^**^	1
2014	PSII	1		
	AFB_1_	0.11	1	
	AFB_2_	0.11	0.85^**^	1
2015	PSII	1		
	AFB_1_	0.13	1	
	AFB_2_	0.11	0.91^**^	1

*Abbreviations* see Table 1, ^**^Correlation is significant at the *p* < 0.01 level

### Detection of QTLs for resistance to aflatoxin contamination

**Fig. 2:**
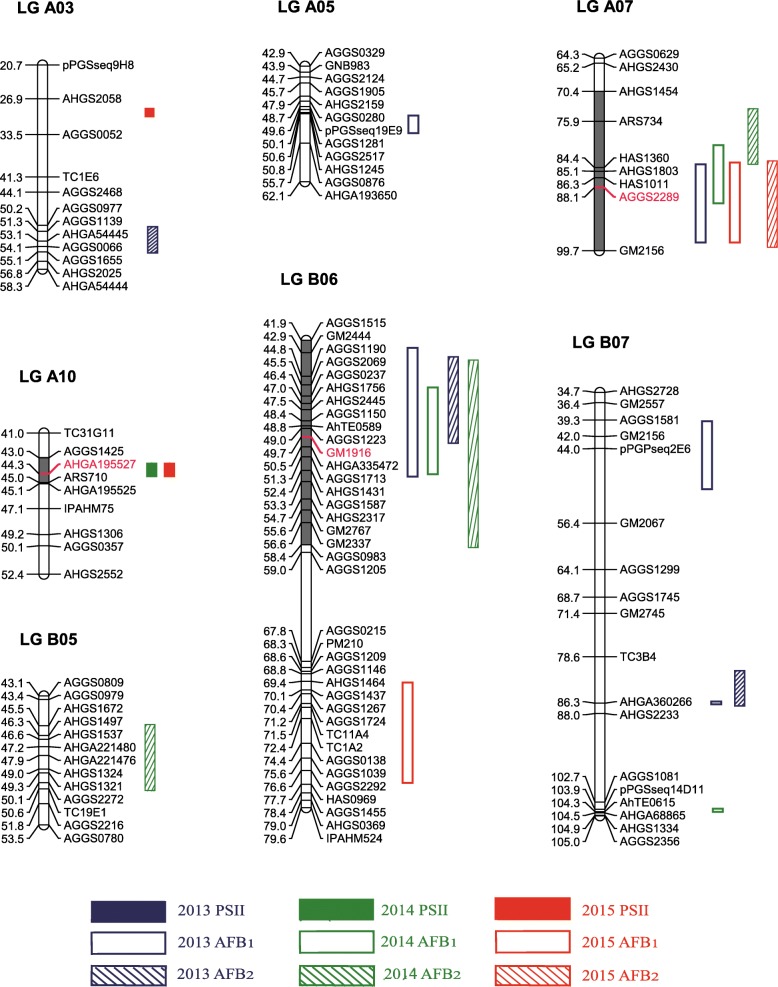
Distribution of QTLs for traits of resistance to aflatoxin contamination on the genetic map. *PSII* percent seed infection index, *AFB*_*1*_ aflatoxin B_1_ content, *AFB*_*2*_ aflatoxin B_2_ content. SSR markers in red color are markers closest to the peak of QTL confidence intervals

Genome-wide QTL analysis was conducted using the high-density genetic map [13] and the phenotypic data of PSII, AFB_1_ and AFB_2_ contents obtained from the RILs during 3 years (2013, 2014 and 2015) in Wuhan. For resistance to aflatoxin contamination, a total of 20 QTLs were identified in three environments that explained 7.30–21.02% PVE (Fig. 2, Table 3). If QTLs for a particular trait were detected on the same genomic region in two or more than two environments, they were considered as one consistent QTL and designated with the same name. Therefore, the 20 QTLs were designated as two for PSII, seven for AFB_1_ and five for AFB_2_ (Table 3). These QTLs were mapped onto seven LGs, comprising four LGs of the A sub-genome and three LGs of the B sub-genome (Fig. 2, Table 3). A maximum of four QTLs were identified onto LG B07, followed by three QTLs onto LG B06 (Fig. 2, Table 3). Two QTLs each were mapped onto LG A03 and A07, as well as one QTL each onto LG A05, A10 and B05.

**Table 3:** QTLs identified for resistance to aflatoxin contamination in the RIL populations across three environments

Trait	QTL^a^	LG^b^	Env^c^	CI^d^	Marker Interval	LOD	PVE (%)^e^	Additive^f^
PSII	*qPSIIA03*	A03	2015	28.50–30.20	AHGS2058 - AGGS0052	3.06	7.96	−2.62
	***qPSIIA10***	A10	2014	43.50–44.70	AGGS1425 – ARS710	5.00	13.00	5.27
			2015	43.70–44.30	AGGS1425 – ARS710	4.40	11.32	3.08
AFB_1_	*qAFB1A05*	A05	2013	51.10–55.70	AHGS1245 - AGGS0876	3.17	7.98	36.02
	***qAFB1A07***	A07	2013	83.40–99.20	ARS734 - GM2156	5.50	14.57	49.00
			2014	80.30–91.00	ARS734 - GM2156	5.98	17.87	35.96
			2015	83.80–98.20	ARS734 - GM2156	4.70	10.62	25.68
	***qAFB1B06.1***	B06	2013	42.50–52.90	AGGS1515 - AGGS1587	6.40	16.33	−52.07
			2014	45.70–52.70	AGGS2069 - AGGS1587	3.90	9.52	−26.31
	*qAFB1B06.2*	B06	2015	69.50–77.60	AHGS1464 - HAS0969	3.11	7.78	−19.13
	*qAFB1B07.1*	B07	2013	39.20–51.70	AGGS1581 - GM2067	3.60	8.48	−40.35
	*qAFB1B07.2*	B07	2013	86.00–86.50	TC3B4 - AHGS2233	3.10	7.30	−36.16
	*qAFB1B07.3*	B07	2014	103.70–104.30	AGGS1081 - AhTE0615	3.20	7.46	−22.55
AFB_2_	*qAFB2A03*	A03	2013	50.19–55.08	AGGS1139 - AHGS2025	3.45	8.32	3.44
	***qAFB2A07***	A07	2014	74.30–84.40	AHGS1454 - HAS1360	3.96	10.84	2.95
			2015	83.50–98.20	ARS734 - GM2156	5.10	12.19	2.20
	*qAFB2B05*	B05	2014	45.40–50.40	AGGS0979 - TC19E1	4.90	11.05	−3.49
	***qAFB2B06***	B06	2013	43.10–50.10	GM2444 - AHGA335472	3.80	9.32	− 3.53
			2014	43.20–58.30	GM2444 - AGGS0983	8.80	21.02	−4.11
	*qAFB2B07*	B07	2013	80.80–86.50	TC3B4 - AHGS2233	5.30	14.45	−4.48

*Abbreviations* see Table 1, ^*a*^ QTLs identified in more than one environment were highlighted in bold, ^*b*^ Linkage group, ^*c*^ Environment, ^*d*^ Confidence interval of QTLs, ^*e*^ The percentage of the phenotypic variation explained, ^*f*^ Additive value

There were two QTLs for PSII across three environments (Table 3). Major QTL *qPSIIA10* was identified as a consistent QTL with 11.32–13.00% PVE, because it was repeatedly detected in two environments (2014 and 2015). Minor QTL *qPSIIA3c* was only detected in 2014 (Table 3).

For AFB_1_ content, a total of seven QTLs were detected comprising two major QTLs and five minor QTLs, with PVE ranging from 7.30 to 17.87% (Table 3). Major QTL *qAFB1A07* was identified across all the three environments and explained 10.62–17.87% PVE (Table 3). The other major QTL *qAFB1B06.1* was detected in two environments with 9.52–16.33% PVE. Minor QTLs namely *qAFB1A05*, *qAFB1B06.2*, *qAFB1B07.1*, *qAFB1B07.2* and *qAFB1B07.3* were only detected in single environment (Table 3).

For AFB_2_ content, five QTLs were detected with a range of 8.32 to 21.02% PVE, including four major QTLs and one minor QTL (Table 3). Major QTLs namely *qAFB2A07* and *qAFB2B06* were consistently detected in two environments and showed 10.84–12.19% and 9.32–21.02% PVE, respectively. But other two major QTLs, *qAFB2B05* and *qAFB2B07,* were only detected in single environment with 11.05–14.45% PVE in addition to minor QTL *qAFB2A03* (Table 3).

Notably, QTLs *qAFB1A07* and *qAFB2A07* were co-localized into the same genetic interval (CI: 74.30–99.20) on LG A07. Similarly, *qAFB1B06.1* was also co-localized with *qAFB2B06* on LG B06 (CI: 43.10–58.30) (Fig. 2, Table 3). These results indicated that each of the two co-localized intervals may simultaneously regulate the resistance to AFB_1_ and AFB_2_ accumulation.

### Conditional QTL mapping

For the purpose of investigating the relationship between QTLs for AFB_1_ and AFB_2_, conditional QTL analyses were performed with conditional phenotypic values y(AFB_1_|AFB_2_) and y(AFB_2_|AFB_1_). Seven QTLs for AFB_1_ were identified in unconditional analysis, whereas six of them failed to be detected when AFB_1_ content was conditioned on AFB_2_ content (Table 4). Major QTL *qAFB1A07* was only detected in 2015, while it was not found in 2013 and 2014 in conditional mapping. Three additional QTLs (*qAFB1A08*, *qAFB1B01*, *qAFB1B08*) were identified with 12.62–15.19% PVE in conditional QTL analysis, but they were only detected in single environment. Of the five QTLs for AFB_2_ identified in unconditional mapping, three were not detected when AFB_2_ content was conditioned on AFB_1_ content (Table 4). Major QTL *qAFB2B06* was detected in 2014 with decreased additive effects (16.74% PVE) compared to its corresponding unconditional QTL (21.02% PVE), however it was not detected in 2013. Minor QTL *qAFB2A03* exhibited slightly enhanced additive effect (9.42% PVE) compared to that of the unconditional QTL (8.32% PVE). Overall, major QTLs for AFB_1_ *qAFB1A07* and *qAFB1B06.1* were severely affected by AFB_2_ content. Similarly, major QTLs for AFB_2_ *qAFB2A07* and *qAFB2B06* were strongly influenced by AFB_1_ content. These results indicated a strong interaction between resistance to AFB_1_ and AFB_2_ accumulation.

**Table 4:** Unconditional and conditional QTLs for aflatoxin accumulation in the RIL population

Condition	QTL	Environment	Marker interval	Unconditional QTL PVE (%)^a^	Conditional QTL PVE (%)^b^
AFB_1_|AFB_2_^c^	*qAFB1A05*	2013	AHGS1245 - AGGS0876	7.98^e^	
	*qAFB1A07*	2013	ARS734 - GM2156	14.57^e^	
		2014	ARS734 - GM2156	17.87^e^	
		2015	ARS734 - GM2156	10.62	9.14^f^
	*qAFB1B06.1*	2013	AGGS1515 - AGGS1587	16.33^e^	
		2014	AGGS2069 - AGGS1587	9.52^e^	
	*qAFB1B06.2*	2015	AHGS1464 - HAS0969	7.78^e^	
	*qAFB1B07.1*	2013	AGGS1581 - GM2067	8.48^e^	
	*qAFB1B07.2*	2013	TC3B4 - AHGS2233	7.30^e^	
	*qAFB1B07.3*	2014	AGGS1081 - AhTE0615	7.46^e^	
	*qAFB1A08*	2014	TC9B8 - AHGA316376		12.62^g^
	*qAFB1B01*	2014	AGGS2497 - AHGA159068		13.44^g^
	*qAFB1B08*	2015	AGGS1664 - AGGS0189		15.19^g^
AFB_2_|AFB_1_^d^	*qAFB2A03*	2013	AGGS1139 - AHGS2025	8.32	9.42^f^
	*qAFB2A07*	2014	AHGS1454 - HAS1360	10.84^e^	
		2015	ARS734 - GM2156	12.19^e^	
	*qAFB2B05*	2014	AGGS0979 - TC19E1	11.05^e^	
	*qAFB2B06*	2013	GM2444 - AHGA335472	9.32^e^	
		2014	GM2444 - AGGS0983	21.02	16.74^f^
	*qAFB2B07*	2013	TC3B4 - AHGS2233	14.45^e^	

*Abbreviations* see Table 1, ^*a*^ The percentage of the phenotypic variation explained by additive effect of unconditional QTL, ^*b*^ The percentage of the phenotypic variation explained by additive effect of conditional QTL, ^*c*^ AFB_1_ conditioned on AFB_2_, ^*d*^AFB_2_ conditioned on AFB_1_, ^*e*^ The unconditional QTL could not be detected in conditional analysis, ^*f*^ The conditional QTL with increased or decreased PVE% to the unconditional QTL, ^*g*^ The additional QTL identified in conditional analysis

### Identification of markers and recombination of QTLs

To evaluate the effect of combined effect of major QTLs *qAFB1A07* and *qAFB1B06.1*, co-dominant SSR markers AGGS2289 (CI: 88.14) and GM1916 (CI: 49.65) were selected as they were located closest to the peaks of QTLs *qAFB1A07* and *qAFB1B06.1* in multiple environments, respectively (see Additional file 2). The genotypes of AGGS2289 and GM1916 derived from Zhonghua 10 were designated as “AA” and “BB”, while the genotypes from ICG 12625 were designated as “aa” and “bb”, respectively. Genotypes of *qAFB1A07* and *qAFB1B06.1* in the RIL population were investigated using these two markers. As shown in Table 5, seeds with genotype AAbb accumulated significantly higher AFB_1_ and AFB_2_ than seeds with other genotypes (aabb, AABB and aaBB) after inoculation with *A. flavus* in all environments, indicating that introgression of any of these two QTLs could raise resistance to aflatoxin accumulation. Moreover, AFB_1_ content in seeds with aaBB genotype (77.97 μg/g) was significantly less than that in seeds with aabb genotype (104.31 μg/g) in 2015, which was the same genotype from resistant parent ICG 12625. Furthermore, there was significantly less AFB_2_ content in seeds with aaBB genotype (6.24 μg/g) compared to that in seeds with aabb genotype (12.02 μg/g) in 2014 (Table 5). Combination of resistant alleles of *qAFB1A07* and *qAFB1B06.1* enhanced the resistance to aflatoxin accumulation in the RIL population. Two elite RILs namely QT0393 and QT0469 exhibited superiority over parents were selected due to less AFB_1_ and AFB_2_ (Table 6). Both of them were identified as aaBB genotype, which means that the elite lines simultaneously possessed the resistant alleles of *qAFB1A07* and *qAFB1B06.1* (Table 6). All the results suggested that SSR markers, AGGS2289 and GM1916, could be applied in genotyping of *qAFB1A07* and *qAFB1B06.1*, and the resistance to aflatoxin accumulation could be improved via combining resistant alleles of these two QTLs.

**Table 5:** Phenotypic effect of QTLs *qAFB1A07*, *qAFB1B06.1* and *qPSIIA10* in the RIL population

Trait	Genotype	2013	2014	2015
AFB_1_ (μg/g)	AAbb	298.83 ± 77.38^a^	196.24 ± 86.95^a^	156.56 ± 57.84^a^
	aabb	181.64 ± 76.88^b^	134.69 ± 46.89^b^	104.31 ± 52.32^b^
	AABB	180.15 ± 77.38^b^	119.60 ± 59.92^b^	84.87 ± 40.91^bc^
	aaBB	147.67 ± 75.99^b^	102.16 ± 55.41^b^	77.97 ± 40.91^c^
AFB_2_ (μg/g)	AAbb	26.10 ± 12.79^a^	19.63 ± 9.19^a^	13.23 ± 5.34^a^
	aabb	17.98 ± 9.05^b^	12.02 ± 6.06^b^	7.78 ± 5.12^b^
	AABB	16.44 ± 8.62^b^	8.91 ± 5.28^bc^	7.75 ± 5.33^b^
	aaBB	14.04 ± 7.73^b^	6.24 ± 3.97^c^	5.56 ± 3.15^b^
PSII (%)	DD	81.79 ± 12.96^a^	70.97 ± 14.98^a^	92.85 ± 6.79^a^
	dd	77.73 ± 27.11^a^	62.86 ± 12.71^b^	87.73 ± 10.19^b^

*Abbreviations* see Table 1, *Genotype* the genotype of RIL lines, *AA* genotype of SSR marker AGGS2289 from Zhonghua 10, *aa* genotype of SSR marker AGGS2289 from ICG12625, *BB* genotype of SSR marker GM1916 from Zhonghua 10, *bb* genotype of SSR marker GM1916 from ICG 12625, *DD* genotype of SSR marker AHGA195525 from Zhonghua 10, *dd* genotype of SSR marker AHGA195527 from ICG 12625; *a,b,c* and *d* means followed by different letter are statistically different at p < 0.05 based on ANOVA and Tamhane’s T2 multiple-comparison

**Table 6:** Resistance to aflatoxin contamination of best RIL lines in three environments

Code	Genotype	AFB_1_ (μg/g)			AFB_2_ (μg/g)			PSII (%)		
		2013	2014	2015	2013	2014	2015	2013	2014	2015
Zhonghua 10	AABBDD	144.10 ± 35.10	143.06 ± 17.07	133.58 ± 14.14	7.60 ± 0.48	8.33 ± 0.35	7.63 ± 0.60	92.33 ± 1.89	93.00 ± 2.16	93.00 ± 1.41
ICG 12625	aabbdd	85.39 ± 9.77	77.56 ± 4.43	86.52 ± 16.99	6.40 ± 1.48	6.59 ± 0.59	6.12 ± 0.93	70.00 ± 3.74	77.67 ± 0.47	73.33 ± 2.49
QT0393	aaBB	55.50 ± 4.95^*^	56.56 ± 4.06	76.51 ± 5.87	4.72	2.66 ± 0.31^**^	4.32 ± 0.32			
QT0469	aaBB	82.87 ± 1.69	77.31 ± 0.76	37.25 ± 2.91^*^	5.84	4.53 ± 0.76	1.58 ± 0.28^**^			
QT0351	dd							70.00 ± 16.33	49.72 ± 4.30^**^	65.55 ± 2.07^*^
QT0451	dd							55.83 ± 2.04^**^	48.33 ± 0.47^**^	66.11 ± 2.83

*Abbreviations* see Table 1 and Table 5, *Code* Code of RIL line, *Difference is significant at *p* < 0.05 level, **Difference is significant at *p* < 0.01 level

SSR marker AHGA195527 (CI: 44.25) was selected for genotyping of *qPSIIA10*, as it was closest marker to the peak of this QTL (see Additional file 2). The genotype derived from Zhonghua 10 was designated as “DD”, and that from ICG 12625 was designated as “dd”. After genotyping of *qPSIIA10* in the RIL population, it was found that PSII of seeds with dd genotype was significantly lower than that of seeds with DD genotype across all the environments (Table 5). Similarly, two RIL lines namely QT0351 and QT0451 with higher resistance to fungal invasion also harbored the dd genotype of *qPSIIA10* (Table 6). These results indicated that introgression of the QTL *qPSIIA10* could enhance the resistance to PSII in peanut.

### Validation of markers

In order to estimate the precision of SSR markers, AGGS2289 and GM1916, these markers were used to profile the Chinese mini-mini core collection of peanut germplasm. A total of 99 accessions were genotyped with AGGS2289 and GM1916 markers. As a result, six accessions were found to possess AAbb genotype and four accessions with aaBB (Table 7). These ten accessions were inoculated with *A. flavus* in laboratory, and aflatoxins contents were detected after 7 days incubation. Both AFB_1_ and AFB_2_ contents in lines with aaBB genotype (68.97 μg/g of AFB_1_ and 8.23 μg/g of AFB_2_) were significantly less than those with AAbb genotype (239.95 μg/g of AFB_1_ and 20.48 μg/g of AFB_2_) (Table 7). In particular, the accession Zh.h1498 only accumulated 26.35 μg/g of AFB_1_ and 1.88 μg/g of AFB_2_), which might be an excellent resource for improving the resistance to aflatoxin accumulation. Therefore, these two validated markers would be potentially very useful in identifying breeding lines with resistance to aflatoxin accumulation.

**Table 7:** Accessions identified by markers AGGS2289 and GM1916 in Chinese mini-mini core collection of peanut germplasm

Genotype	Code	AFB_1_ (μg/g)	AFB_2_ (μg/g)
AAbb	Zh.h1507	459.69 ± 27.17	38.64 ± 1.39
	Zh.h4809	264.78 ± 7.21	20.52 ± 2.43
	Zh.h3364	210.92 ± 32.26	15.16 ± 0.55
	Zh.h3216	184.12 ± 31.32	19.12 ± 0.46
	Zh.h6275	166.66 ± 8.53	14.54 ± 2.95
	Zh.h3689	153.51 ± 26.28	14.94 ± 3.62
	Mean	239.95 ± 104.65^*^	20.48 ± 8.43^*^
aaBB	Zh.h1498	26.35 ± 1.43	1.88 ± 0.60
	Zh.h2193	86.37 ± 12.32	11.56 ± 4.70
	Zh.h2888	80.32 ± 4.43	10.64 ± 4.90
	Zh.h6070	82.86 ± 1.47	11.21 ± 5.62
	Mean	68.97 ± 24.70	8.23 ± 4.02

*Abbreviations* see Table 1 and Table 5, *Code* Code of Chinese mini-mini core collection of peanut germplasm, ^*^Difference is significant at *p* < 0.05 level, ^**^Difference is significant at *p* < 0.01 level

## Discussion

Aflatoxin contamination is a global challenge for peanut industry and consumers. Genetic enhancement for resistance to aflatoxin is regarded as the most cost-effective approach to reduce contamination risk in this crop. There are several reports on peanut resistance to aflatoxin contamination, but most of them are using transcriptome and proteome analysis to reveal the mechanism of resistance [26–31]. This study is the first systematic report using linkage analysis to reveal the QTLs for two types of resistance i.e., resistance to *A. flavus* infection and aflatoxin accumulation in peanut based on multi-environment phenotyping. Although there was only one previous study on QTL mapping for resistance to fungal invasion which was conducted using the phenotyping data generated for single environment [20]. Six QTLs related to *Aspergillus flavus* invasion were identified in their study, but the specific position information of these QTLs was not provided. We still mapped our markers of resistance to *A. flavus* infection on A10 to their QTLs, but none of them was located in QTLs they identified. Realizing the complexity of the trait, the current study was designed for generating multi-environment phenotyping data which, upon analysis, detected two QTLs for fungal invasion and 12 QTLs for aflatoxin accumulation were identified. The multi-environment phenotyping data allowed to identify one major QTL for PSII which was consistently detected on LG A10 in two environments. Coincidentally, in RNA-seq analysis of peanut seeds infected by *A. flavus*, relative abundance of expression of genes was significantly higher in pseudomolecule A10 [29]. Two consistent and major QTLs for AFB_1_ were identified on LG A07 and LG B06. Similarly, two consistent and major QTLs were also detected for AFB_2_ onto the same genetic intervals of LG A07 and LG B06. Identification of these QTLs started to lift the veil of genetic mechanism controlling resistance to aflatoxin contamination in peanut.

### Resistance to fungal infection and aflatoxin accumulation found independent to each other

So far, there were very few reports on the relationship between resistances to *A. flavus* infection and aflatoxin accumulation in peanut. A previous report stated no significant relationship between these two resistance mechanisms and inherited independently [32]. Another report observed very low correlation and indicated to be governed by different genes [33]. Recently, an *A. flavus* strain with green fluorescent protein (GFP)-expression was used to monitor fungal growth by infection of ten peanut lines. No direct correlation was found between fungal infection and aflatoxin accumulation, which revealed that aflatoxin accumulation depended on genotypes of seeds but not *A. flavus* fungal growth [34]. In present study, no significant correlation was observed between resistance to fungal infection and to aflatoxin accumulation by inoculation in laboratory (Table 2). Furthermore, the major QTL for PSII was mapped on LG A10, while the major QTLs for AFB_1_ and AFB_2_ were identified on LG A07 and LG B06 (Fig. 2). Major QTLs for resistance to fungal invasion and to aflatoxin accumulation were distributed on different chromosomes. Although one minor QTL for PSII and one minor QTL for AFB_2_ were identified on LG A03, they were located at the different genetic intervals (Table 3). These results further confirmed that resistance to fungal infection and to aflatoxin accumulation in peanut were independent of each other. The similar phenomenon was also observed in other studies [32, 33]. As mycotoxins are produced by complex secondary metabolic pathways of fungal, and fungal infections could be affected by carbon sources, nitrogen sources and secondary metabolites of host plants. For example, ethylene in host plant could affects the colonization and infection of *Aspergillus flavus* but not aflatoxin production in maize [35]. Peanut seeds which are resistant to fungal infection but still accumulate large amounts of aflatoxins may because that their host environments are more conducive to toxin production.

### Strong interaction detected between two major QTLs for AFB_1_ and AFB_2_

Significant positive correlation was detected between AFB_1_ and AFB_2_ contents (Table 2). It was interesting to note that the one major QTL each for AFB_1_ (*qAFB1A07*) and AFB_2_ (*qAFB2A07)* were found co-localized on LG A07. Similarly, the QTL, *qAFB1B06.1* for AFB_1_ was also found co-localized with the QTL *qAFB2B06* for AFB_2_ on LG B06 (Fig. 2, Table 3). Additionally, conditional QTL analysis indicated that when AFB_1_ content was conditioned on AFB_2_ content, *qAFB1A07* and *qAFB1B06.1* failed to be detected in 2013 and 2014 (Table 4). Even in 2015, *qAFB1A07* was detected but with decreased additive effects compared to its corresponding unconditional QTL (Table 4). When AFB_2_ content was conditioned on AFB_1_ content, *qAFB2A07* was unable to be detected in all environments (Table 4). The QTL, *qAFB2B06,* was absent in 2013, but present in 2014 with reduced PVE (Table 4). These results indicated strong interaction between AFB_1_ and AFB_2_ contents in peanut, and resistance to AFB_1_ and AFB_2_ may be controlled by the same genomic regions/genes. Hence, it is possible to simultaneously improve the peanut resistance to AFB_1_ and AFB_2_ accumulation.

### Combination of major QTLs *qAFB1A07* and *qAFB1B06.1* provides effective strategy for improving resistance to aflatoxin contamination

Major QTLs *qAFB1A07* and *qAFB1B06.1* were additively interacted with each other. In the RIL population, introgression of any resistant allele could improve the resistance to aflatoxin accumulation (Table 5). When resistant alleles of these two QTLs were combined together, RILs accumulated less aflatoxin compared to those with single resistant allele of *qAFB1A07* (Table 5). Moreover, RIL lines, QT0393 and QT0469, which accumulated less aflatoxin compared to resistant parent ICG 12525, had both the resistant alleles from these two QTLs (Table 6). Additionally, in the Chinese mini-mini core collection, accessions with both resistant alleles accumulated significantly less aflatoxins compared to accessions without any of them (Table 7). The above results suggest that combination of the resistant alleles of *qAFB1A07* and *qAFB1B06.1* is an effective strategy for improving resistance to aflatoxin contamination in peanut.

It is worth mentioning that no significant difference was observed between aflatoxin contents in RILs with both resistant alleles and those with single resistant allele of *qAFB1B06.1* (Table 5), because aflatoxin accumulation could be easily affected by multiple factors, even in controlled laboratory conditions [36, 37]. There was also no significant difference between aflatoxin contents in RILs with *qAFB1A07* and them in RILs with *qAFB1B06.1* (Table 5). But the resistant parent ICG 12625, which only possessed resistant allele of *qAFB1A07*, accumulated significantly less aflatoxin compared to the susceptible parent Zhonghua 10 which only had resistant allele of *qAFB1B06.1* (Table 1), implying that there must be additional genes responsible for the resistance in ICG 12625.

### Linked SSR markers, AGGS2289 and GM1916, exhibited potential deployment in molecular breeding for improving aflatoxin resistance

Linked SSR markers namely AGGS2289 and GM1916 were used to genotype *qAFB1A07* and *qAFB1B06.1*, from which, four peanut resistant accessions were successfully identified in the Chinese mini-mini core collection (Table 7). SSR markers, AGGS2289 and GM1916, identified in this study could be used in improving aflatoxin resistance breeding. In addition, more breeder-friendly markers linked to QTLs *qAFB1A07* and *qAFB1B06.1* would be developed in the future to facilitate breeding for resistance to aflatoxin contamination.

## Conclusions

The present study identified one major QTL for resistance to PSII and two important co-localized intervals associated with major QTLs for resistance to AFB_1_ and AFB_2_. Combination of these intervals could improve the resistance to aflatoxin accumulation in peanut. SSR markers linked to these intervals were identified and validated. The major QTLs, co-localized intervals and SSR markers identified in this study showed great value for improvement of resistance to aflatoxin contamination in peanut. Additionally, this study laid the foundation for revealing genetic basis of resistance to aflatoxin contamination and further research on fine mapping and candidate gene discovery.

## Additional files

Additional file 1: Analysis of variance for PSII, AFB_1_ and AFB_2_ in the RIL population across three environments. (XLSX 10 kb)

Additional file 2: Position and sequences of diagnostic markers. (XLSX 8 kb)

## Abbreviations

AFB_1_: Contents of aflatoxin B1
AFB_2_: Contents of aflatoxin B2
LGs: Linkage groups
PSII: Percent seed infection index
PVE: Phenotypic variance explained
QTL: Quantitative trait loci
RILs: Recombinant inbred lines

## References

[1] Food and agriculture organization of the united nations (FAO): Production/Yield quantities of groundnuts, with shell in world. http://www.fao.org/faostat/en/#data/QC/visualize (2016). Accessed 28 May 2018.

[2] Settaluri VS, Kandala CVK, Puppala N, Sundaram J (2012). Peanuts and their nutritional aspects-a review. Food Nutr Sci.

[3] Kew MC (2013). Aflatoxins as a cause of hepatocellular carcinoma. J Gastrointestin Liver Dis.

[4] Khlangwiset P, Wu F (2010). Costs and efficacy of public health interventions to reduce aflatoxin-induced human disease. Food Addit Contam Part A Chem Anal Control Expo Risk Assess..

[5] Patten RC (1981). Aflatoxins and disease. Am J Trop Med Hyg.

[6] Sugri I, Osiru M, Abudulai M, Abubakari M, Asieku Y, Lamini S, Zakaria M (2017). Integrated peanut aflatoxin management for increase income and nutrition in northern Ghana. Cogent Food Agriculture.

[7] Al-Saad L (2017). Biotic and abiotic control of aflatoxin b1 synthesis. Riga: Noor publishing.

[8] Torres AM, Barros GG, Palacios SA, Chulze SN, Battilani P (2014). Review on pre- and post-harvest management of peanuts to minimize aflatoxin contamination. Food Res Int.

[9] Dorner JW (2008). Management and prevention of mycotoxins in peanuts. Food Addit Contam Part A Chem Anal Control Expo Risk Assess.

[10] Horn BW, Dorner JW (1998). Soil populations of *Aspergillus* species from section Flavi along a transect through peanut-growing regions of the United States. Mycologia..

[11] Chulze S, Palazzini JM, Torres AM, Barros G, Ponsone ML, Geisen R (2015). Biological control as a strategy to reduce the impact of mycotoxins in peanuts, grapes and cereals in Argentina. Food Addit. Contam. Part A Chem. Anal. Control Expo. Risk Assess.

[12] Bhatnagarmathur P, Sunkara S, Bhatnagarpanwar M, Waliyar F, Sharma KK (2015). Biotechnological advances for combating *Aspergillus flavus* and aflatoxin contamination in crops. Plant Sci.

[13] Huang L, Ren X, Wu B, Li X, Chen W, Zhou X (2016). Development and deployment of a high-density linkage map identified quantitative trait loci for plant height in peanut (*Arachis hypogaea* L.). Sci Rep.

[14] Chen Y, Ren X, Zheng Y, Zhou X, Huang L, Yan L (2017). Genetic mapping of yield traits using RIL population derived from Fuchuan Dahuasheng and ICG6375 of peanut (*Arachis hypogaea* L.). Mol Breed.

[15] Luo H, Guo J, Ren X, Chen W, Huang L, Zhou X (2017). Chromosomes A07 and A05 associated with stable and major QTLs for pod weight and size in cultivated peanut ( *Arachis hypogaea L*.). Theor Appl Genet.

[16] Ravi K, Vadez V, Isobe S, Mir RR, Guo Y, Nigam SN (2011). Identification of several small main-effect QTLs and a large number of epistatic QTLs for drought tolerance related traits in groundnut (*Arachis hypogaea* L.). Theor Appl Genet.

[17] Zongo A, Khera P, Sawadogo M, Shasidhar Y, Sriswathi M, Vishwakarma MK (2017). SSR markers associated to early leaf spot disease resistance through selective genotyping and single marker analysis in groundnut (*Arachis hypogaea* L.). Biotechnol Rep (Amst).

[18] Zhao Y, Zhang C, Chen H, Yuan M, Nipper R, Prakash CS (2016). QTL mapping for bacterial wilt resistance in peanut (*Arachis hypogaea* L.). Mol Breed.

[19] Varshney RK, Pandey MK, Janila P, Nigam SN, Sudini H, Gowda MVC (2014). Marker-assisted introgression of a QTL region to improve rust resistance in three elite and popular varieties of peanut (*Arachis hypogaea* L.). Theor Appl Genet.

[20] Liang X, Zhou G, Hong Y, Chen X, Liu H, Li S (2009). Overview of research progress on peanut (*Arachis hypogaea* L.) host resistance to aflatoxin contamination and genomics at the Guangdong academy of agricultural sciences. Peanut Science.

[21] Asis R, Muller V, Barrionuevo DL, Araujo SA, Aldao MA (2009). Analysis of protease activity in *Aspergillus flavus* and A. *parasiticus* on peanut seed infection and aflatoxin contamination. Eur J Plant Pathol.

[22] Wang H, Huang J, Lei Y, Yan L, Wang S, Jiang H (2013). Relationship of resveratrol content and resistance to aflatoxin accumulation caused by *Aspergillus flavus* in peanut seeds. Acta Agron Sin.

[23] IBM Corp. Statistical package for social scineces (IBM SPSS) 22.0 version. Armonk: IBM United States; 2013. https://www.ibm.com/analytics/data-science/predictive-analytics/spss-statistical-software. Accessed 25 May 2018.

[24] Wang S, Basten C. Windows QTL Cartographer 2.5. Department of Statistics, North Carolina State University, Raleigh, NC. 2012. http://statgen.ncsu.edu/qtlcart/WQTLCart.htm. Accessed 01 May 2018.

[25] Zhu J (1995). Analysis of conditional genetic effects and variance components in developmental genetics. Genetics..

[26] Korani W, Chu Y, Holbrook CC, Oziasakins P (2018). Insight into genes regulating post-harvest aflatoxin contamination of tetraploid peanut from transcriptional profiling. Genetics..

[27] Fountain JC, Jin K, Yang L, Pandey MK, Nayak SN, Bajaj P, et al. Proteome analysis of *Aspergillus flavus* isolate-specific responses to oxidative stress in relationship to aflatoxin production capability. Sci Rep. 2018;8(1):3430. 10.1038/s41598-018-21653-x PMC5821837 29467403

[28] Fountain JC, Yang ML, Pandey MK, Nayak S, Kumar V, Jayale A, et al. RNAseq analysis reveals oxidative stress responses of *Aspergillus flavus* are related to stress tolerance and aflatoxin production. In: American Phytopathological society annual meeting. American Phytopathological Society. 2016. https://www.apsnet.org/meetings/Documents/2016_meeting_abstracts/aps2016_74.htm. Accessed 25 May 2018.

[29] Nayak SN, Agarwal G, Pandey MK, Sudini HK, Jayale AS, Purohit S (2017). *Aspergillus flavus* infection triggered immune responses and host-pathogen cross-talks in groundnut during in vitro seed colonization. Sci Rep.

[30] Wang H, Lei Y, Wan L, Yan L, Lv J, Dai X (2016). Comparative transcript profiling of resistant and susceptible peanut post-harvest seeds in response to aflatoxin production by *Aspergillus flavus*. BMC Plant Biol.

[31] Wang H, Lei Y, Yan L, Wan L, Ren X, Chen S (2016). Functional genomic analysis of *Aspergillus flavus* interacting with resistant and susceptible peanut. Toxins..

[32] Utomo SD, Anderson WF, Wynne JC, Beute MK, Hagler WM, Payne GA (1990). Estimates of heritability and correlation among three mechanisms of resistance to *Asperaillus parasiticus* in peanut. American Peanut Research and Education Society Meeting.

[33] Upadhyaya HD, Nigam SN, Thakur RP. Genetic enhancement for resistance to aflatoxin contamination in groundnut. In: The seventh ICRISAT regional groundnut meeting for Western and Central Africa: International Crops Research Institute for the Semi-Arid Tropics, Haderabad, India; 2002.

[34] Korani WA, Chu Y, Holbrook C, Clevenger J, Ozias-Akins P (2017). Genotypic regulation of aflatoxin accumulation but not *Aspergillus* fungal growth upon post-harvest infection of peanut (Arachis hypogaea L.) seeds. Toxins..

[35] Wang S, Park Y-S, Yang Y, Borrego EJ, Isakeit T, Gao X, Kolomiets MV (2017). Seed-derived ethylene facilitates colonization but not aflatoxin production by *Aspergillus flavus* in maize. Front Plant Sci.

[36] Payne GA, Brown MP (1998). Genetics and physiology of aflatoxin biosynthesis. Annu Rev Phytopathol.

[37] Guo B, Chen Z, Dewey LR, Brian TS (2008). Drought stress and preharvest aflatoxin contamination in agricultural commodity: genetics, genomics and proteomics. J Integr Plant Biol.

